# Supplementary material for: Does repeatedly viewing overweight versus underweight images change perception of and satisfaction with own body size?
Source: R Soc Open Sci. 2020 Apr 1;7(4):190704. doi: 10.1098/rsos.190704 (PMC7211892; doi:10.1098/rsos.190704)
Supplement: Further information on Implicit Measures [file rsos190704supp1.docx]

**Supplementary Material from “Does repeatedly viewing overweight versus underweight images change satisfaction with own body size?”**

**Further details of the Implicit Tasks described in the paper**

Participants completed a series of novel experimental measures designed to capture possible changes in their implicit beliefs about their body size and satisfaction with body size. These are described in more detail here.

- - 1. ***Lexical Decision Task***

Participants were primed to their own body by completing the Avatar “own body” task, in which they were asked to make the avatar their own size and shape. They then completed the Lexical Decision Task described by Smith (1). The task is to respond as quickly as possible regarding whether the groups of letters presented are real words or not. The task contains real words in the categories of “beauty”, “ugly”, “neutral” and “positive”. Reaction times were calculated for “beauty” minus “ugly” words, and for “neutral” minus “positive” words. The LDT task was presented in ePrime.

- - 1. ***Lexical Decision Task – Recall***

Following two further tasks and a five-minute break, participants were given one minute to write down as many of the real words from the LDT as they were able to recall. The number of “beauty” minus number of “ugly” words, and number of “neutral” minus number of “positive” words recalled were calculated.

- - 1. ***Implicit Association Test – Words***

The Implicit Association Test (IAT) (2) is one of the most frequently used tasks to assess implicit cognitions. It seeks to “measure implicit attitudes by measuring their underlying automatic evaluation” (2). Participants are asked to sort “target” words and “attribute” words using different keys on the computer keyboard. The theory is that reaction times will be quicker if the words which are sorted using the same key are “evaluatively compatible” or “congruent” than if they are non-compatible (or incongruent).

For the task to work, it is necessary that the words be as similar as possible to each other in terms of length, number of syllables, and familiarity. For this study, it was also important that the words were viewed as being positive or negative with regard to weight and shape. There is no database indicating the age at which people generally learn words associated with weight and shape, so we designed a brief online questionnaire in order to select and characterise such words.

Participants were presented with 19 words chosen because they were related to weight and shape (toned, fit, slender, slim, trim, lean, lithe, curvaceous, shapely, thin, skinny, obese, fat, flabby, chunky, tubby, podgy, stout, chubby and plump). For each word, they were asked “How negative or positive is this word when used to describe a woman’s body?” with scoring options from 1 (very negative) to 6 (very positive). For each word, they were then asked how old they thought they were when they first learnt it.

The questionnaire ran online from 3^rd^ July 2015 to 24^th^ July 2015, and we received 164 responses from 18-30 year old women with English as their first language. Using their responses to these two questions, and the characteristics of the words in terms of length and number of syllables, positive and negative body words were matched on length, estimated age of acquisition, and median score (“positive” words scored a median of 5 or 6; “negative” words scored a median of 1 or 2).

The study was approved by the Oxford University Central University Research Ethics Committee (MS-IDREC-C1-2015-123).

Following the results from this study, we chose the following “Attribute” words relating to the concept of “Slim”: trim, slim, fit, slender; the “Attribute” words related to “Not Slim” were: stout, podgy, obese, fat. Participants were asked to sort words belonging to the “Target” categories of Self (me, mine, I, myself) and Other (she, they, their, her).

After a brief practice trial using words relating to trees, flowers, cities and countries, participants completed a practice trial for Self/Slim pairings, and then a longer trial of this pairing, followed by a practice and then longer trial of “Self/Not Slim” pairings. The task was conducted using ePrime.

- - 1. ***Implicit Association Task – Images***

For this task, the same methodology as the IAT word task described above was used, but words relating to body size were replaced with images of the same women modified to appear of normal weight or slightly overweight (selected from the images in the *Morphed Photographic Figure Scale* (3)). The task was conducted using ePrime.

1. Smith AR, Joiner TE, Jr., Dodd DR. Examining implicit attitudes toward emaciation and thinness in anorexia nervosa. The International journal of eating disorders. 2014;47(2):138-47.

2. Greenwald AG, McGhee DE, Schwartz JL. Measuring individual differences in implicit cognition: the implicit association test. J Pers Soc Psychol. 1998;74(6):1464-80.

3. The Morphed Photographic Figure Scale: Creation and validation of a novel set of realistic female body stimuli [Internet]. OSF. 2017 [cited 22.3.19].
